# Severe Pulmonary Disease Caused by *Mycolicibacter kumamotonensis*

**DOI:** 10.3201/eid2703.191648

**Published:** 2021-03

**Authors:** Katerina Manika, Fanourios Kontos, Apostolos Papavasileiou, Dimitrios Papaventsis, Maria Sionidou, Ioannis Kioumis

**Affiliations:** G. Papanikolaou Hospital, Thessaloniki, Greece (K. Manika, M. Sionidou, I. Kioumis);; Attikon University Hospital, Athens, Greece (F. Kontos);; Sotiria Chest Diseases Hospital, Athens (A. Papavasileiou, D. Papaventsis)

**Keywords:** pulmonary disease, nontuberculous mycobacteria, *Mycolicibacter kumamotonensis*, bacteria, respiratory infections, Greece, tuberculosis and other mycobacteria

## Abstract

Severe *Mycolicibacter kumamotonensis-*pulmonary disease was diagnosed in a 68-year-old immunocompetent woman in Greece; the disease was initially treated as tuberculosis. The patient responded favorably to a new treatment regimen of azithromycin, amikacin, moxifloxacin, and linezolid. Complete symptom resolution and radiologic improvement resulted.

Species belonging to genus *Mycolicibacter* (formerly *Mycobacterium terrae complex*) are considered not pathogenic, with the exception of causing chronic tenosynovitis of the hand (*1*,*2*). We present a case of severe pulmonary disease caused by *Mycolicibacter kumamotonensis*, a pathogen that was described in 2006 (*3*).

A 68-year-old woman in Greece had had shortness of breath, productive cough, and low-grade fever for several weeks. The patient was from Georgia but had been living in Greece for the preceding 20 years; she had a history of breast cancer, which had been treated with chemotherapy and radiotherapy 7 years earlier, and bronchiectasis. During the preceding 3 years, the patient had recurrent chest infections and received multiple antimicrobial drug regimens. Based on a positive sputum acid-fast staining, standard antituberculosis treatment was initiated. Culture of the sputum sample was macroscopically suggestive of nontuberculous mycobacteria, but identification of the species was not feasible because of poor growth and technical problems. After 1 month the patient reported improvement of her symptoms and total resolution of fever. Her erythrocyte sedimentation rate (ESR) dropped from 46 to 25 mm/h (reference range 0–20 mm/h), and her weight was stable. Computed tomography (CT) scan of her chest showed multiple cavities, bronchiectasis, nodules, and tree-in-bud appearance (Figure, panels A–C). Bronchoscopy was performed, but PCR for *Μycobacterium tuberculosis*, acid-fast stain, and culture of the bronchial washing were all negative.

**Figure:**
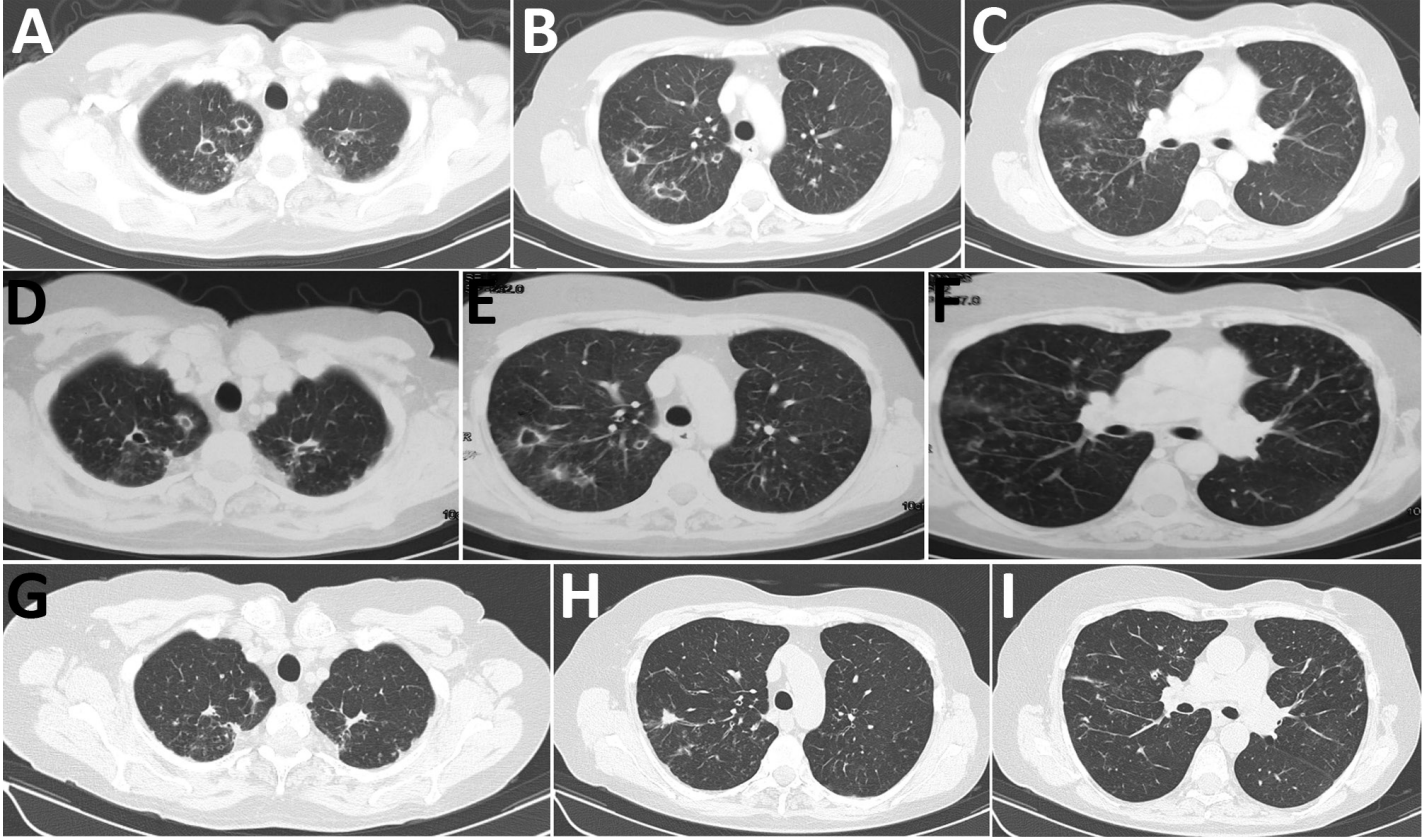
Chest computed tomography scan 1 month after antituberculosis treatment initiation (A–C), at the time of *Mycolicibacter kumamotonensis* identification (D–F), and 1 year after treatment initiation for *M. kumamotonensis* (G–I). Resolution of cavities and scar formation (A to D to G), resolution of pulmonary infiltrations (B to E to H), and hardening of the nodular appearances (C to F to I) are shown.

Five months into treatment, the patient’s condition gradually worsened. She developed productive cough and shortness of breath with hypoxemia (SpO_2_ of 91% breathing room air), and her ESR rose to 59 mm/h. A new bronchoscopy was performed. Acid-fast staining results were negative, whereas results of a culture on MGIT960 automated system (strain GR- 21075) (Becton Dickinson, http://www.bd.com) and Lowenstein-Jensen slants (bioMérieux, https://www.biomerieux.com) were positive. No other pathogens were isolated.

For molecular identification, we sequenced regions of 927 bp of 16S rDNA gene and of 440 bp of the 65-kDa heat shock protein (hsp65) gene (3730 DNA analyzer; Applied Biosystems, https://www.thermofisher.com) using the Big Dye Terminator Cycle Sequencing Kit (Applied Biosystems) and previously described primers (*4*). We compared sequences with those of validly published species in the National Center for Biotechnology Information (http://www.ncbi.nlm.nih.gov) using BLAST (http://hsp65blast.phsa.ca/blast/blast.html) and deposited them in GenBank (accession nos. MT491187 and MT491188).

The sequence of 16S rDNA and hsp65 genes showed 100% similarity with the type strain of *Mycolicibacter kumamotonensis* (strain CST7274). We then determined the MICs (SLOMYCOI; TREK Diagnostic Systems, http://www.trekds.com) (*5*). We found the strain had susceptibility to clarithromycin (MIC 1 μg/mL), amikacin (MIC 16 μg/mL), doxycycline (MIC ≤0.12 μg/mL), rifabutin (MIC ≤0.25 μg/mL), ethambutol (MIC 2 μg/mL), and trimethoprim/sulfamethoxazole (MIC 2/38 μg/mL); intermediate susceptibility to linezolid (MIC 16 μg/mL); and resistance to rifampin (MIC >8 μg/mL), ciprofloxacin (MIC 8 μg/mL), and moxifloxacin (MIC 4 μg/mL).

At the end of antituberculosis treatment, a second CT scan revealed slight improvement of the nodules and the tree-in-bud appearance but persistence of the cavities (Figure, panels D–F). Because of the patient’s clinical deterioration and the isolation of *M. kumamotonensis* from bronchoalveolar lavage (*1*), we initiated treatment with azithromycin (500 mg 5 d/wk), amikacin (750 mg intramuscular, 5 d/wk), moxifloxacin (400 mg), and linezolid (600 mg). The patient reported complete resolution of symptoms and gained 2 kg of bodyweight, and her ESR dropped to 15 mm/h. One year after diagnosis, a new CT scan showed further improvement, with closure of cavities (Figure, panels G–I). However, many of the nodules persisted. The patient is now fully active and working. The plan is to continue treatment for another 6 months.

*M. kumamotonensis* has been isolated from respiratory specimens, lymph nodes, and soft tissue all over the world (*3*,*4*,*6*,*7*). Most of these reports, however, do not include data on the clinical implications of *M. kumamotonensis* identification. In their recent report, Iemura-Kashiwagi et al. (*7*) describe the case of soft tissue infection successfully treated with a combination of antimicrobial drugs and surgical debridement. Compared with that report, the MICs of our strain were higher for most of the drugs, possibly because of our patient’s history of chest infections treated with multiple regimens.

In an older study, Smith et al. (*8*) reported that 14 out of 54 patients with *M. terrae* infection had pulmonary disease. Because *M. kumamotonensis* and *M. arupense* are the most frequently isolated species of the complex (*9*), some of these cases could in fact be attributed to *M. kumamotonensis.* On the other hand, *M. kumamotonensis* has recently been found in a hospital environment (*10*), so laboratory contamination of clinical specimens is a possibility. Based on the complete resolution of symptoms and the improvement after the appropriate treatment was initiated, we do not consider contamination to be the case with our patient.

The patient responded favorably to the selected regimen even though the strain was resistant to moxifloxacin and of borderline MIC to linezolid. Increase of moxifloxacin dose was not attempted because of fear of QT prolongation in an elderly woman. In conclusion, *M. kumamotonensis* infection should be included in the differential diagnosis of mycobacterial pulmonary disease with cavity formation in immunocompetent adults with bronchiectasis.
